# A Twenty-Year Retrospective Cohort Study of Mortality and Morbidities in Adult Trauma Patients with Blunt, Sharp, and Firearm Injuries

**DOI:** 10.3390/medicina62020235

**Published:** 2026-01-23

**Authors:** Sophia Rosella Lee, Aaron Wang Lee, Michael J. Erickson, Steven E. Wolf, Juquan Song

**Affiliations:** 1 John Sealy School of Medicine, University of Texas Medical Branch, Galveston, TX 77555, USA; 2 Department of Surgery, University of Texas Medical Branch, Galveston, TX 77555, USAjusong@utmb.edu (J.S.)

**Keywords:** traumatic injury, mortality, morbidity, blood transfusions, shock

## Abstract

*Background and Objectives*: Traumatic injuries are a major public health issue, being the leading cause of death in the U.S. Advancements in medical care, injury prevention, and regional trauma systems have improved survival rates, but there is limited information on outcomes for survivors. Blunt, sharp, and firearm injuries are the primary mechanisms in trauma forensics. This study examines patient outcomes for blunt, sharp, and firearm injuries over 20 years. *Materials and Methods*: De-identified data were collected from the TriNetX Research network in June 2024. Patients aged 18–90 were categorized by injury type (blunt, sharp, firearm) from 2004 to 2023. Trends were analyzed by stratifying the data into 20 consecutive one-year intervals. Mortality, blood transfusions, traumatic shock, hypovolemic shock, and acute post-hemorrhagic anemia were recorded annually. Statistical analysis was performed using One Way Repeated ANOVA and post hoc Tukey testing, with significance defined as *p* < 0.05. *Results*: The study included 1,205,350 blunt, 710,875 sharp, and 144,562 firearm injuries. Firearm injuries predominantly affected males (83%) and African Americans (51%), while blunt and sharp injuries showed more demographic variability. Looking at the 20-year trends, the average age of firearm and sharp injury patients decreased by 21% (48 ± 13 to 38 ± 15, *p* ≤ 0.0001) and 14% (49 ± 16 to 42 ± 18, *p* ≤ 0.0001), respectively, while blunt injury patient age did not change significantly. Mortality rates significantly decreased from 12% for firearm, 7% for sharp, and 6% for blunt injuries in 2004 to less than 1% in 2023 for all three injury mechanisms. Blood transfusions increased 450% (2% to 11%) for firearm injuries and increased 100% for sharp and blunt injuries (1% to 2%). Traumatic shock and hypovolemic shock incidences also increased by 100% for firearm injuries (3% to 6% and 1% to 2%, respectively), while sharp and blunt injuries did not change significantly. Acute post-hemorrhagic anemia increased from 3% to 19% for firearm injuries (533% relative increase), while sharp and blunt injuries remained around 3% for the past 20 years. *Conclusions*: The study reveals that with improved survival rates over the last 20 years, there has been a significant increase in shock-related morbidities and blood transfusion rates, particularly for firearm injuries. These findings can inform trauma care to enhance resuscitation efforts, optimize resource allocation, and improve mortality and outcomes for these injury mechanisms.

## 1. Introduction

Traumatic injuries represent a significant public health concern as they are the leading cause of death in the U.S. for people under the age of 45, regardless of demographic or socioeconomic factors [1]. Nationally, traumatic injury is the third leading cause of death across all age groups, claiming 227,039 lives in 2022 [1]. The toll of these injuries extends beyond mortality, with an estimated loss of 25.42 million potential years of life in the last five years and a staggering healthcare burden amounting to $4.71 trillion in 2020 alone [2].

Within the field of trauma forensics, firearm, sharp, and blunt injuries are the most common fatal mechanisms [3]. In 2020, firearms were the leading mechanism of fatal injury, causing 35,346 deaths, followed by sharp injury (2550 deaths), and blunt injury (1365 deaths). When compared to the overall population, firearm injuries have a much higher crude rate (5.66), followed by sharp (0.64) and blunt (0.42) mechanisms. In contrast, other mechanisms like drowning (0.01), poisoning (0.03), and burns (0.03) occur at much lower crude rates [3]. In addition to mortality, morbidities following trauma create a large burden on the healthcare system. Over the last five years, these three trauma mechanisms have collectively contributed to the loss of almost six million potential years of life and have accumulated healthcare costs exceeding $866 billion [4,5].

Advancements in medical care, coupled with injury prevention strategies and the establishment of regional trauma systems, have significantly enhanced survival rates among trauma patients [6]. Despite these strides, recent national data suggest that the burden of violent injury among survivors is growing more complex. For example, a recent U.S. study analyzing nearly one million patients with firearm or sharp traumatic injuries from 2010 to 2019 reported a significant rise in firearm-related injuries and demonstrated that violent trauma is associated with substantially higher hospitalization costs, shorter intervals to readmission, and greater odds of non-home discharge [7]. These findings highlight that the outcomes and morbidities of these patients warrant further investigation. This study aims to explore patient outcomes with three initial types of traumas (firearm, sharp, and blunt injury) over a 20-year period by using TriNetX, a large national database. We hypothesize that there is an increased survival of trauma victims over the past twenty years associated with a rise in post-trauma morbidities.

## 2. Materials and Methods

**Figure 1:**
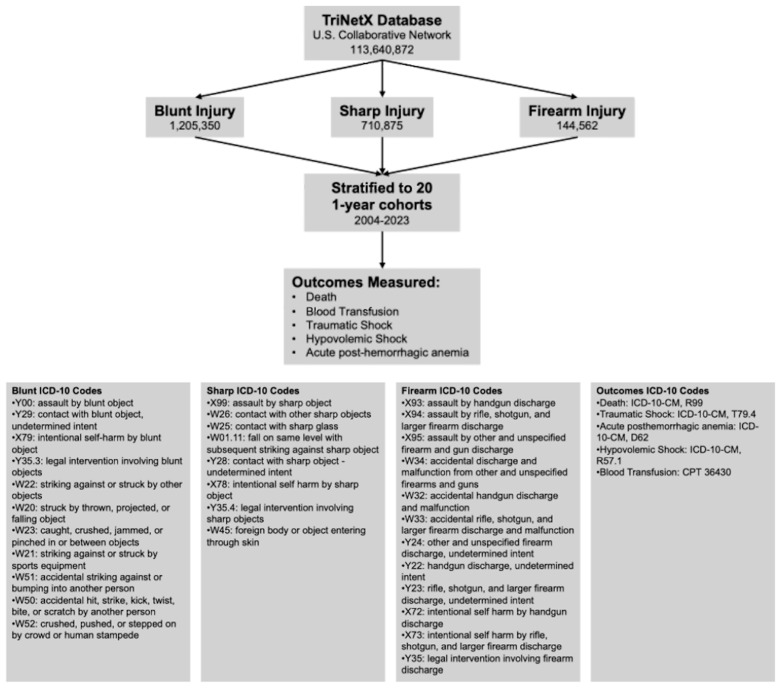
Flowchart of the TriNetX cohorts, outcomes measures, and ICD-10 codes for the years 2004–2023.

Patients’ de-identified data were acquired from TriNetX Research network (TriNetX LLC, Cambridge, MA, USA) in June 2024. Adult patients aged 18–90 years were eligible for inclusion if they had at least one ICD-10 diagnosis code corresponding to blunt, sharp, or firearm injury recorded between 1 January 2004, and 31 December 2023. Patients were excluded only if they did not meet the age criterion or lacked trauma-related ICD-10 codes. Blunt, sharp, and firearm injuries were defined using pre-specified ICD-10 code sets based on mechanism of injury. Blunt injuries included codes indicating non-penetrating force mechanisms; sharp injuries included codes for lacerations and penetrating trauma from sharp objects; and firearm injuries included codes describing injuries from projectile discharge of any firearm. The complete list of ICD-10 codes and their grouping is presented in Figure 1.

To evaluate changes over the two-decade period, three separate queries, one for blunt injuries, one for sharp injuries, and one for firearm injuries, were executed independently for each calendar year from 2004 through 2023, resulting in 60 total queries. All outcomes were measured one year following this index event. Because of platform limitations, patients could appear in more than one annual cohort if they sustained qualifying injuries in different years, and they could also contribute to more than one trauma mechanism across years. Additionally, because patient identities cannot be deduplicated across years, the study characterizes annual trauma events rather than unique individuals.

Data on mortality, blood transfusions, traumatic shock, hypovolemic shock, and acute post-hemorrhagic anemia were recorded in Microsoft Excel (version 16.90) (Figure 1). Post-traumatic shock was chosen in addition to hypovolemic shock; its ICD-10 code specifically looks at shock secondary to traumatic injuries, whereas the ICD-10 code for hypovolemic shock encompasses all shock states caused by a blood or fluid volume depletion, regardless of etiology.

Descriptive statistics and figures were generated using GraphPad Prism version 10.2.3. One-Way Repeated Analysis of Variance (ANOVA) and post hoc Tukey Test (*t*-test) were performed for comparison and significance. Statistical significance was defined as a *p*-value less than or equal to 0.05. TriNetX is HIPAA and GDPR compliant and the study does not include Protected Health Information or Personal Data. The study was pre-approved by IRB (IRB # 20-0085) on 16 April 2020 as the electronic patient record data are de-identified and aggregated.

## 3. Results

### 3.1. Patient Population

A total of 1,205,350 patients with blunt injury, 710,875 with sharp injury, and 144,562 with firearm injury were included in this study from 1 January 2004 to 31 December 2023 (20 years). The average age for blunt was 42 ± 19, sharp was 44 ± 18, and firearm was 40 ± 15. For sex composition, males were the largest percentage of all three mechanisms of injury. Among female patients, blunt injury was the most common. Regarding racial composition, White patients accounted for the largest proportion of blunt and sharp injury, whereas Black patients constituted the largest proportion of firearm injury. For ethnicity, non-Hispanic/Latino patients comprised the majority across all injury types. When analyzing geographic distribution across U.S. regions, the Northeast had the largest proportion of blunt injury, while the South had the largest proportion of sharp and firearm injury (Table 1).

### 3.2. Twenty-Year Trends of Patient Demographics

**Figure 2:**
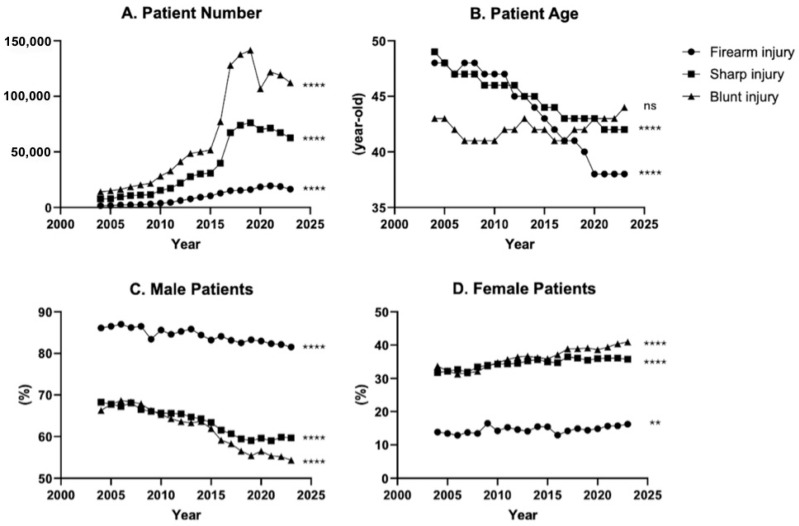
20-years of blunt, sharp, and firearm injury patient number, age, and sex from 2004 to 2023. (**A**) Number of patients, (**B**) average age of patient in years, (**C**) Male patient % composition, (**D**) female patient % composition. ** *p* ≤ 0.01, **** *p* ≤ 0.0001.

Over the past two decades, there has been an increase in all three types of traumatic injury when stratified into 20 consecutive one-year intervals. However, in the most recent three years (2021–2023), there has been a decline in trauma patients for all three mechanisms, with firearm decreasing by 15%, sharp by 13%, and blunt injury by 8% (Figure 2A). Interestingly, the average age of patients decreased for both firearm and sharp injury over the past two decades, while remaining stable for blunt injury. Firearm injury age decreased by 21% and sharp injury age by 14% (Figure 2B).

While males continued to represent the majority of traumatic injuries, there was an upward trend of female trauma patients observed, most notably for blunt injury. Female firearm injuries have increased by 17%, sharp injuries by 12.7%, and blunt injuries by 21.6% over the past 20 years (Figure 2C,D).

**Figure 3:**
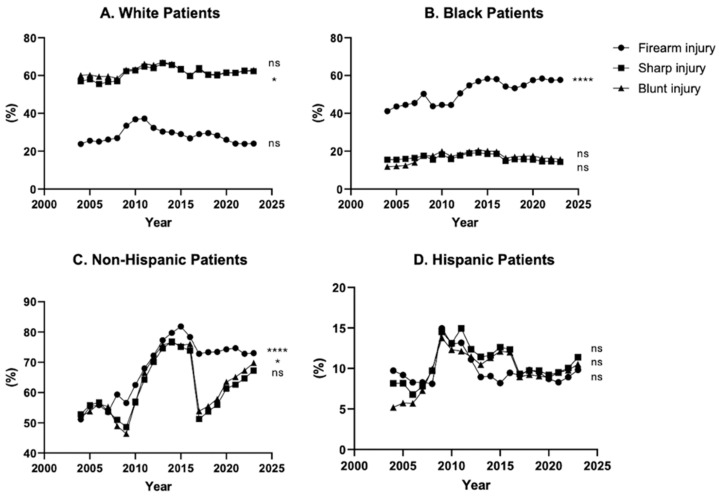
20 years of blunt, sharp, and firearm injury patient race and ethnicity from 2004 to 2023. (**A**) White patient % composition, (**B**) Black patient % composition, (**C**) Non-Hispanic patient % composition, (**D**) Hispanic patient % composition. * *p* ≤ 0.05, **** *p* ≤ 0.0001.

Analyzing racial demographics, there was a statistically significant 40% increase in Black firearm injuries, while White firearm injuries have remained stable over the past two decades. Conversely, sharp injuries showed a statistically significant 9.4% increase in White patients with no significant change observed among Black patients. Notably, blunt injury showed no significant change for either racial group over the same period (Figure 3A,B). For all three injury types, non-Hispanic/Latino patients have increased to compose approximately 70% in 2023, while Hispanic/Latino patients compose 10% of traumas. Over the entire two-decade span, non-Hispanic/Latino patients demonstrated a statistically significant 43% increase in firearm and 34% increase in blunt injury, while Hispanic/Latino patients showed no significant change across all injury types (Figure 3C,D).

**Figure 4:**
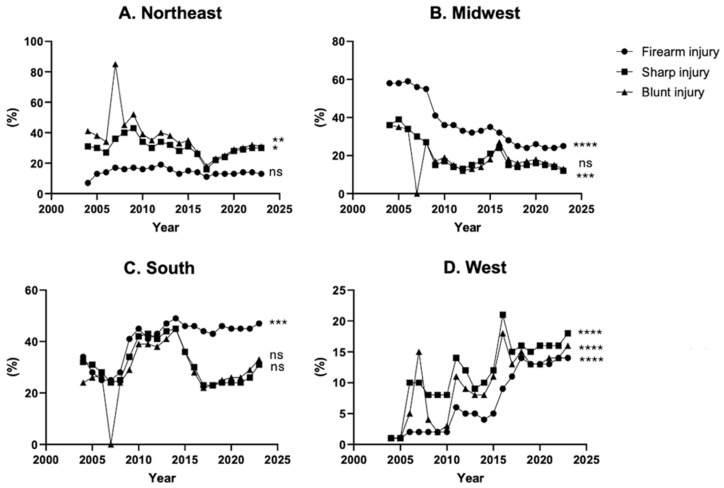
20 years of blunt, sharp, and firearm injury patient percent composition by US regions from 2004 to 2023. * *p* ≤ 0.05, ** *p* ≤ 0.01, *** *p* ≤ 0.001, **** *p* ≤ 0.0001.

Examining regional trends within the United States, blunt injury decreased by 24% in the Northeast, decreased by 64% in the Midwest, increased by 64% in the South, and increased by 38% in the West over the past two decades. For sharp injury, there was a 67% decrease in the Midwest and a 1700% increase in the West, while remaining stable in the Northeast and South. All regions had a statistically significant change for firearm injuries with the Northeast increasing by 86%, the Midwest decreasing by 57%, the South increasing by 38%, and the West increasing by 1300% (Figure 4A–D).

### 3.3. Twenty-Year Trends of Patient Measured Outcomes

**Figure 5:**
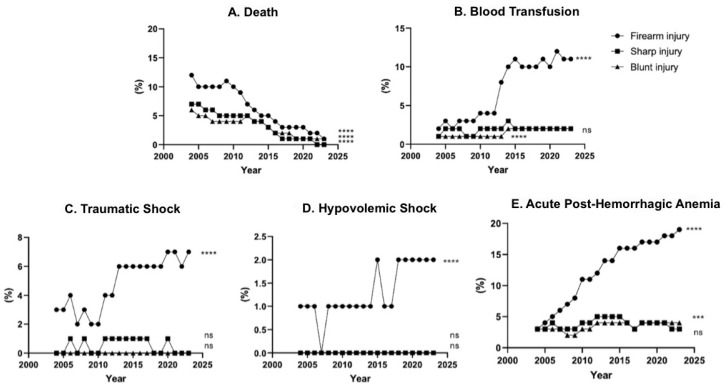
20-years of blunt, sharp, and firearm injury patient percent incidence of mortality, morbidity, and blood transfusions from 2004 to 2023. *** *p* ≤ 0.001, **** *p* ≤ 0.0001.

The mortality rates among trauma patients significantly decreased from 12% for firearm, 7% for sharp, and 6% for blunt injury in 2004 to 1% or less in 2023 (Figure 5A). Furthermore, the incidence of blood transfusions increased markedly over the past two decades, with firearm injury seeing a 5.5-fold increase, and sharp and blunt injury doubling. (Figure 5B) For traumatic shock, there was a 2-fold increase for firearm injury, while there was no significant change observed for blunt and sharp injury (Figure 5C). Similarly, hypovolemic shock also doubled for firearm injury, with no significant changes noted for blunt and sharp injury (Figure 5D). Lastly, acute posthemorrhagic anemia exhibited a notable 6-fold increase for firearm injury, whereas sharp and blunt injury showed no significant change (Figure 5E).

## 4. Discussion

This study provides a comprehensive analysis of trauma patterns over the past two decades, focusing on blunt, sharp, and firearm injury. The findings reveal significant trends in patient demographics, geographic distributions, mortality, morbidities, and patient care, contributing valuable insights into trauma care.

### 4.1. Demographic Insights

Our study indicates that the average ages of patients with blunt, sharp, and firearm injury are relatively similar. However, the decreasing average age of firearm and sharp injury patients over the past two decades indicates a troubling trend toward younger individuals being affected. This shift underscores the urgent need for preventive measures targeting youth, including education, community outreach, and policy interventions. DiMaggio et al. (2016) explored trauma patient populations and found an increase in age between 2000 and 2011, the opposite of what our study found [6]. When analyzing our data from 2004 to 2011, there was no significant difference in the average age. However, examining the period from 2011 to 2023 revealed a significant decrease in age that was not evident before 2011. In line with our findings, Tomas et al. (2023) found an increase in firearm injuries for patients aged 25–44 between the years 2012 to 2019 [8].

The higher prevalence of males in all injury categories aligns with existing literature on trauma demographics. Notably, firearm injuries are the most common among male patients, whereas blunt injuries predominate among female patients. Gani et al. (2017) found that from 2006 to 2014, males had a 9-fold higher incidence of firearm related accidental and intentional injuries compared to female patients [9]. Consistent with our findings, Tomas et al. (2023) noted a significant increase in the proportion of female trauma patients, particularly in cases involving sharp, blunt, and firearm injuries between 2012 and 2019 [8]. This disparity suggests gender-specific risk factors and behaviors that warrant targeted prevention efforts. The upward trend of female trauma patients, especially in blunt injuries, suggests a potentially increased exposure to injury risks among women in the recent two decades.

The racial composition highlights distinct patterns, with White patients predominantly experiencing blunt and sharp injury, while Black patients are more frequently affected by firearm injury. These findings underscore the need for culturally sensitive interventions and community-based strategies to address the unique challenges faced by different racial groups. Fowler et al. (2015) found that Black Americans had the highest rate of firearm mortality overall (14.8 per 100,000), approximately 10-fold higher than White Americans (1.4 per 100,000) between 2010 and 2012 [10]. This trend underscores the persistent risk of firearm-related deaths within Black communities. Similarly, Kalesan et al. (2017) found an increase in firearm injury rates for Black Americans between 2001 and 2013 that was 4-fold greater than that of White Americans [11].

Geographic analysis reveals notable regional differences in trauma patterns. The Northeast’s higher prevalence of blunt injury contrasts with the South’s higher rates of sharp and firearm injury. Elkbuli et al. (2020) found a similar trend of higher rates of penetrating injuries in the South compared to other regions in the National Trauma Data Bank [12]. Additionally, DiMaggio et al. (2016) found a statistically significant increase in trauma injuries for both the South and West from 2000 to 2011 [6]. Of note, the South and West have lower trauma surgeon density and percentage of Level I trauma centers compared to the Northeast and Midwest [13]. These variations suggest that regional socioeconomic factors, availability of healthcare resources, and local policies influence trauma incidence. Importantly, the magnitude of trauma pattern changes in the West, particularly the 1700% increase in sharp injuries and 1300% increase in firearm injuries, is striking. While these findings highlight a rapidly evolving regional burden, the underlying drivers of these dramatic increases remain unclear and warrant further investigation. Understanding the factors that contribute to these disproportionate rises, including socioeconomic conditions, state-level legislation, and access to firearms, will be key to developing effective region-specific interventions and resource allocation strategies.

### 4.2. Mortality and Morbidities Insights

Over the past two decades, there has been an increase in all three types of injury, with firearm injury exhibiting the most significant rise. However, the recent decline in sharp (−18%) and blunt (−21%) trauma cases since 2019, is encouraging. Additionally, the substantial decrease in mortality rates across all injury types is a testament to advancements in trauma care and emergency medical services. However, the increased incidence of blood transfusions and shock-related complications, particularly in firearm injuries, indicates ongoing challenges in managing severe trauma cases.

The decrease in trauma patient mortality can be attributed to a combination of systematic improvements in trauma care protocols, emergency response efficiency, and advanced medical interventions. In line with our findings, studies by Dutton et al. (2010) and Kaufman et al. (2022) found a significant improvement in trauma patient mortality in the 21st century [14,15]. Additionally, Negoi et al. (2015) observed a progressive, continuous decrease in trauma mortality rates rather than a trimodal distribution, consistent with our study [16]. Furthermore, Patel et al. (2011) and Havermans et al. (2020) demonstrated that optimizing trauma protocols significantly enhances survival rates [17,18]. Collectively, these studies underscore the critical impact of advanced patient care on reducing mortality rates in recent decades.

In pre-hospital trauma care, Williamson et al. (2011) highlights several key improvements [19]. One major advancement is the use of supraglottic airway devices and video laryngoscopy, which improve the ability to secure airways in challenging environments [19]. Hemorrhage control has also improved significantly with the introduction of new hemostatic agents and tourniquets, which have become standard practice for managing severe hemorrhage in the field [19]. Furthermore, the implementation of structured trauma systems, particularly regional trauma networks, has optimized the transport of patients to appropriate trauma centers, reducing time to definitive care [19]. These advancements, along with increased emphasis on rapid transport and early intervention, have contributed to improved survival rates and outcomes for trauma patients in the prehospital setting.

Blood transfusion practices in trauma patients have evolved significantly over recent years, driven by research emphasizing their critical role in improving patient outcomes. Cushing et al. (2011) showed that early and increased use of blood components such as platelets, fibrinogen, and plasma enhance survival rates among severely injured patients [20]. Likewise, Inaba et al. (2010) found that this shift towards more aggressive plasma usage, even in patients not requiring massive transfusion, correlates with better outcomes [21]. This shift in blood transfusion protocol can explain the steep increase in blood transfusions for trauma patients reflected in this study after 2012.

Goel et al. (2021) similarly found in national trauma databases that blood transfusions occurred in 12.7% of U.S. firearm injury hospitalizations [22]. Additionally, they found that major/extreme severity of illness for firearm injuries was correlated with a higher prevalence of transfusions [22]. In this study, the firearm injury morbidities of traumatic shock, acute post hemorrhagic anemia, and hypovolemic shock have clearly followed similar trends to firearm injury blood transfusions, increasing by 2-fold, 6-fold, and 2-fold, respectively. The higher incidence of traumatic shock, acute posthemorrhagic anemia, and hypovolemic shock in firearm injury further emphasizes the severity of these injuries. The significant rise in blood transfusion rates for firearm injury reflects the need for robust trauma care systems equipped to manage such critical cases effectively. These findings highlight the importance of continued investment in trauma systems and research to improve patient outcomes.

### 4.3. Limitations

This study had several limitations. First, the resource information was indeterminate as not all the injuries were trauma certified. Second, the geographic distribution within specific regions was broadly assessed, which may have introduced biases and missed contextual factors. Additionally, the codes used to identify injury types may have inadvertently included other injury types. Furthermore, the outcomes of interest could take several years to manifest, yet our study was constrained to one year following the injury.

Finally, the Web-based Injury Statistics Query and Reporting System (WISQARS) from the Center of Disease Control’s (CDC) Injury Center was used to cross-reference the major results of this study. Similarly to the TriNetX database, WISQARS data from 2004 to 2022 showed a higher number of blunt injuries (67,873,192), followed by sharp (34,002,628), and firearm injuries (2,412,319) [23,24,25,26,27,28]. Notably, the mortality rate decreased for firearm injuries from 33% in 2004 to 23% in 2022 in WISQARS, comparable to the 12% to 1% decrease in firearm mortality rate observed between 2004 and 2023 in TriNetX [27,28]. However, there were some differences between the TriNetX and WISQARS findings. For both sharp and blunt injuries, WISQARS data indicated an increase in mortality rates of 1.4-fold and 1.6-fold, respectively, whereas our results reported a significant decrease for both [23,24,25,26]. Despite these discrepancies, particularly concerning sharp and blunt injuries, the similar trends in firearm injury mortality rates between the databases are encouraging.

## 5. Conclusions

This study found that with increased trauma survival, there was an increase in shock-related morbidities and blood transfusion rates for patients. These findings provide critical insights into the evolving landscape of trauma over the past two decades. Understanding this observed increase in shock related morbidities and blood transfusion rates among trauma patients is critical for informing and updating treatment protocols to enhance early resuscitation strategies, optimize resource allocation, and ultimately improve patient outcomes for these three trauma injury mechanisms. Future research should focus on further improving trauma care protocols for acute care as well as investigating the longitudinal recovery, morbidities, and chronic healthcare needs of trauma patients.

## Figures and Tables

**Table 1:** Overall trauma patient population demographics and *p*-values from 2004–2023.

		Blunt Injury	Sharp Injury	Firearm Injury	Firearm vs. Blunt	Firearm vs. Sharp	Sharp vs. Blunt
Patient Population	Number of Patients	1,205,350	710,875	144,562	<0.001	0.004	0.004
Age	Mean ± SD	42 ± 19	44 ± 18	40 ± 15	0.254	0.254	0.004
Sex	Male	58%	61%	83%	<0.001	<0.001	0.139
	Female	38%	36%	15%	<0.001	<0.001	0.139
Ethnicity	Not Hispanic or Latino	63%	62%	69%	0.020	0.001	0.709
	Hispanic or Latino	10%	11%	10%	0.757	0.025	0.003
Race	White	62%	62%	30%	<0.001	<0.001	0.595
	Black	17%	16%	51%	<0.001	<0.001	0.254
U.S. Regions	Northeast	30%	28%	16%	<0.001	0.004	0.004
	Midwest	17%	16%	26%	<0.001	<0.001	1.000
	South	29%	29%	44%	<0.001	<0.001	1.000
	West	13%	15%	11%	<0.001	<0.001	0.009

## Data Availability

Datasets generated and/or analyzed in preparation for the current study are presented in the manuscript and additional information is available from the corresponding author upon reasonable request.

## Abbreviations

The following abbreviations are used in this manuscript:

ANOVA	Analysis of Variance
*t*-test	Tukey Test
WISQARS	Web-based Injury Statistics Query and Reporting System
CDC	Center of Disease Control
